# Clinical Value of the Systemic Immune Inflammation Index and PD-L1 Expression in Advanced NSCLC Treated with Pembrolizumab: Real-World Preliminary Study

**DOI:** 10.32604/or.2026.077514

**Published:** 2026-05-21

**Authors:** Hyungkeun Cha, Yong Seok Lee, Gui Young Kwon, Boran Kim, Yeonsook Moon, Lucia Kim, Hae-Seong Nam

**Affiliations:** 1 Division of Pulmonology, Department of Internal Medicine, Inha University Hospital, Inha University School of Medicine, Incheon, Republic of Korea; 2 Department of Obstetrics and Gynecology, College of Medicine, The Catholic University of Korea, Seoul, Republic of Korea; 3 Pathology Division, Seoul Clinical Laboratories, Yongin-si, Gyeonggi-do, Republic of Korea; 4 Incheon-Namdong Branch, National Health Insurance Corp., Incheon, Republic of Korea; 5 Department of Laboratory Medicine, Inha University Hospital, Inha University School of Medicine, Incheon, Republic of Korea; 6 Department of Pathology, Inha University Hospital, Inha University School of Medicine, Incheon, Republic of Korea

**Keywords:** Systemic immune inflammation index (SII), programmed death-ligand 1 (PD-L1) expression, pembrolizumab, non-small-cell lung cancer (NSCLC), biomarkers

## Abstract

**Objective:** Studies on the comprehensive utility of complete blood count-derived inflammatory biomarkers (CBC-IBs) as biomarkers in pembrolizumab-treated advanced non-small-cell lung cancer (NSCLC) are scarce. This study aimed to investigate the clinical relevance of a panel of CBC-IBs as potential predictive biomarkers and assess whether integrating the systemic immune-inflammation index (SII) with programmed death-ligand 1 (PD-L1) expression could overcome the limitations of PD-L1 as a standalone predictive biomarker. **Methods:** Our real-world preliminary study was conducted on a cohort of patients with advanced NSCLC. Patients who had undergone PD-L1 immunohistochemistry testing at the time of diagnosis, and had completed at least three cycles of pembrolizumab were included. The CBC-IBs analyzed in this study were calculated using absolute cell counts of neutrophils, lymphocytes, monocytes, and platelets. **Results:** A total of 102 patients were included. Low baseline SII was significantly associated with superior progression-free survival (PFS) (*p* = 0.031) and overall survival (OS) (*p* = 0.004). In multivariate analysis, SII emerged as the strongest independent predictor for OS among all evaluated CBC-IBs. Furthermore, patients with a combination of low SII and high PD-L1 expression demonstrated the most favorable survival outcomes. **Conclusion:** Although further prospective and multicenter studies are needed to validate the generalizability of our findings, the clinical implication is that the use of pretreatment SII and/or PD-L1 expression values may predict therapeutic outcomes and assist in optimizing individualized treatment strategies for patients with advanced NSCLC.

## 1 Introduction

Over the more than 100-year history of cancer immunotherapy development, the advent of immune checkpoint inhibitors (ICIs) represents a revolutionary milestone in cancer treatment. Among ICIs, the most successful therapeutic approach involves targeting programmed death-1 (PD-1) or its ligand PD-L1. This strategy has received approval for the treatment of a wide range of malignancies, including non-small-cell lung cancer (NSCLC). Anti-PD-1/PD-L1 therapies are currently the most widely implemented form of immunotherapy and have transformed the oncological therapeutic landscape [1,2].

Pembrolizumab is a highly selective, humanized monoclonal antibody that targets PD-1, inhibiting its interaction with PD-L1 and PD-L2 [3]. It was the first immunotherapeutic agent approved by the U.S. Food and Drug Administration (FDA) for first-line treatment of advanced NSCLC in 2016 and has since gained approval for use in various other tumor types [4,5]. Pembrolizumab is now used as monotherapy or in combination with chemotherapy in advanced NSCLC, depending on the level of PD-L1 expression [6].

Unlike targeted therapies such as tyrosine kinase inhibitors, a major challenge in the clinical application of cancer immunotherapy with anti-PD-1/PD-L1 agents is the lack of reliable predictive biomarkers for response and survival. Although PD-L1 expression in tumor cells has been proposed as a logical biomarker for predicting responses to anti-PD-1/PD-L1 therapies, its predictive value remains limited [7]. A study demonstrated that the objective response rate to pembrolizumab in NSCLC was only 45%, even among patients with high PD-L1 expression levels [3]. These findings emphasize the need for robust, universally applicable, cost-effective biomarkers to predict therapeutic outcomes better and guide clinical decision-making in patients receiving anti-PD-1/PD-L1 therapy.

Inflammation, defined as the host inflammatory response, is now recognized as a hallmark of cancer development and progression [8,9,10]. The functional interplay between inflammation and malignancy has been increasingly elucidated through studies on various systemic inflammatory biomarkers. Of these, the complete blood count (CBC) with differential, which is a routinely performed, cost-effective test in clinical practice, provides valuable insights into the nature and intensity of the host inflammatory response both before and after cancer treatment. CBC-derived inflammatory biomarkers (CBC-IBs), including the systemic immune-inflammation index (SII), absolute neutrophil count (ANC), neutrophil–lymphocyte ratio (NLR), derived NLR, platelet–lymphocyte ratio (PLR), and monocyte–lymphocyte ratio (MLR), reflect the balance of peripheral blood cell populations involved in inflammation and immune regulation; these biomarkers have been associated with predictive and prognostic outcomes in various malignancies [11,12,13,14,15]. However, limited studies have evaluated the comprehensive utility of CBC-IBs as biomarkers in advanced NSCLC patients treated with pembrolizumab. 

Therefore, we investigated the clinical relevance of a panel of CBC-IBs as potential predictive biomarkers for treatment response and survival in this patient population. Additionally, we assessed whether integrating SII, as a potent independent factor, with PD-L1 expression could complement the limitations of PD-L1 expression as a standalone predictive biomarker.

## 2 Materials and Methods

### 2.1 Study Design and Patients

**Figure 1:**
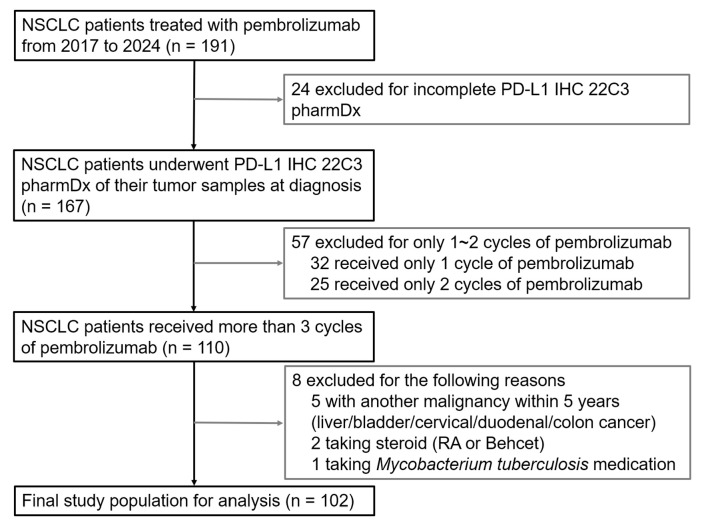
Flowchart illustrating the patient enrollment process for the study cohort. Note: IHC, immunohistochemistry; NSCLC, non-small-cell lung cancer; PD-L1, programmed death ligand 1; RA, rheumatoid arthritis.

We conducted a retrospective cohort study of patients with NSCLC who received pembrolizumab in various clinical settings at INHA university hospital between 2017 and March 2024. Patients aged ≥ 20 years who had histologically confirmed primary NSCLC, had undergone PD-L1 testing using the 22C3 pharmDx immunohistochemistry (IHC) assay at the time of diagnosis, and had completed at least three cycles of pembrolizumab were included. Fig. 1 illustrates the patient enrollment process. Patients were excluded if they had a history of other malignancies within the previous 5 years or were receiving treatment for conditions associated with systemic inflammation (infections, hematologic disorders, or connective tissue diseases), as these may have influenced the CBC-IBs. 

Baseline clinicopathological and laboratory variables at the initiation of pembrolizumab treatment were extracted retrospectively from the electronic medical records system. Patient-related variables included age, sex, smoking status, and Eastern Cooperative Oncology Group performance status (ECOG PS). Laboratory parameters obtained within 2 weeks prior to the first pembrolizumab administration included hemoglobin, albumin, lactate dehydrogenase (LDH), C-reactive protein (CRP), and several CBC-IBs. Tumor-related variables comprised histologic subtype and metastatic stage. All patients were staged according to the eighth edition of the TNM classification system (AJCC/UICC) [16], based on contrast-enhanced chest or abdominal computed tomography (CT), brain magnetic resonance imaging or CT, and positron emission tomography CT (PET-CT) or whole-body bone scan. 

Written informed consent for PD-L1 IHC testing was obtained from all patients prior to undergoing tissue biopsy for lung cancer diagnosis. The study was performed in accordance with the Declaration of Helsinki. The Institutional Review Board of Inha University Hospital approved the study protocol (no. IUH-IRB 202406016). Survival data were obtained from the institutional electronic medical records system and the Korean Ministry of Security and Public Administration.

### 2.2 PD-L1 IHC

The PD-L1 IHC 22C3 pharmDx has been approved by the FDA as a companion diagnostic for pembrolizumab therapy in patients with NSCLC [4]. In the present study, PD-L1 expression was evaluated in NSCLC tumor specimens using the validated PD-L1 IHC 22C3 pharmDx assay (Agilent Technologies/Dako, SK-506, Santa Clara, CA, USA), an *in vitro* diagnostic (IVD)-labeled kit designed for clinical PD-L1 testing. 

Formalin-fixed, paraffin-embedded tumor specimens obtained via diagnostic tissue biopsy were used for analysis. Tissue samples were fixed in 10% neutral buffered formalin (Sigma-Aldrich, HT501128, St. Louis, MO, USA) for 6–48 h prior to routine tissue processing and paraffin embedding. Tissue sections were cut at a thickness of 4 μm using a rotary microtome and mounted on positively charged glass slides. All immunohistochemical staining procedures were conducted at Seoul Clinical Laboratories (Yongin, Republic of Korea).

The 22C3 pharmDx assay was performed using the Autostainer Link 48 (Agilent Technologies/Dako, AS480, Santa Clara, CA, USA) with the manufacturer’s optimized closed protocol for this automated platform [17,18]. Appropriate positive and negative controls were included in each staining run following the manufacturer’s instructions. All stained sections were independently evaluated by two experienced pathologists (G.Y.K and L.K). Any discrepancies in scoring were resolved through consensus after re-examination.

PD-L1 expression was scored using the tumor proportion score (TPS), defined as the percentage of viable tumor cells exhibiting partial or complete membranous PD-L1 staining of any intensity. Tumor cell evaluation was performed by manual microscopic estimation. Cases containing at least 100 viable tumor cells were considered adequate for PD-L1 scoring. The TPS was calculated as the number of PD-L1–positive tumor cells divided by the total number of viable tumor cells and multiplied by 100 [19]. Based on the TPS value, PD-L1 expression was categorized using predefined cut-off values (TPS < 1%, TPS 1–49%, and TPS ≥ 50%).

### 2.3 Definition of CBC-IBs

All patients routinely underwent blood testing, including a CBC, within 2 weeks prior to the initiation of pembrolizumab therapy. The CBC-IBs analyzed in this study were calculated using absolute cell counts of neutrophils, lymphocytes, monocytes, and platelets. The following ratios were derived: neutrophil count/lymphocyte count (NLR), neutrophil count/(white blood cell-neutrophil) count (dNLR), monocyte count/lymphocyte count (MLR), platelet count/lymphocyte count (PLR), and platelet count × NLR (SII). Optimal cutoff values for these biomarkers were determined based on overall survival (OS) using maximally selected rank statistics, calculated with the *maxstat* package in R v4.4.1 (R Core Team, Vienna, Austria) with *p*-values adjusted for multiple testing according to Lausen and Schumacher [20].

### 2.4 Statistical Analysis

Categorical variables were analyzed using the *χ*^2^ test or Fisher’s exact test. To account for multiple comparisons, the Bonferroni correction was applied where appropriate. Continuous variables are presented as medians and ranges, whereas categorical variables are presented as absolute numbers and percentages. Cutoff values for hemoglobin, albumin, LDH, and CRP were determined based on the normal reference ranges established at our institution. Progression-free survival (PFS) was defined as the interval from the date of the first pembrolizumab administration to the date of radiologically confirmed disease progression or death from any cause. Disease progression was assessed through radiological evaluations conducted during the follow-up period according to RECIST v1.1. OS was defined as the time from the first pembrolizumab administration to the date of death or last follow-up. The impact of each clinical factor on survival outcomes was assessed using the Kaplan–Meier method, and comparisons were made using the log-rank test for univariate analysis. Variables exhibiting a statistically significant association (*p* < 0.05) in univariate analysis were subsequently included in a multivariate Cox proportional hazards regression model to evaluate their independent effects. Variable selection for the Cox model was conducted using a forward stepwise approach. The proportional hazards assumption of the Cox model was validated through visual inspection of Kaplan–Meier survival curves and log-log survival plots to ensure they did not cross. The predictive accuracy of each biomarker was assessed by calculating the C-index and time-dependent ROC curves based on the linear predictor (*Xβ*) derived from the Cox proportional hazards model. Hazard ratios (HRs), adjusted for potential confounders, along with 95% confidence intervals (CIs), were reported. The absence of multicollinearity was confirmed using the Variance Inflation Factor (VIF), with all final variables showing VIF < 5. All statistical tests were two-sided, and significance was evaluated at a level of *p* < 0.05. Statistical analyses were performed using SPSS v26.0 (IBM Corp., Armonk, NY, USA).

## 3 Results

### 3.1 Patient Characteristics

In total, 102 patients who had received at least three cycles of pembrolizumab and undergone PD-L1 IHC staining of tumor samples at diagnosis were included in our preliminary study. Baseline characteristics prior to the first cycle of pembrolizumab are summarized in Table 1. The median age of the patients was 69 (46–86) years, with a predominance of males (n = 88; 86.3%). Most patients were former smokers (46.1%) and had an ECOG PS of 1 (65.7%) and adenocarcinoma histology (57.8%). Extrapulmonary metastases were confirmed in 40 patients (39.2%). Of these, brain, liver, bone, and adrenal gland metastases were observed in 27 (26.5%), 7 (6.9%), 33 (32.4%), and 8 (7.8%) patients, respectively. The median serum hemoglobin, albumin, LDH, and CRP levels were 12.0 (7.8–17.9) g/dL, 3.95 (2.2–5.0) g/dL, 217 (133–981) IU/L, and 0.92 (0.04–11.23) mg/dL, respectively. In total, 26 patients (25.5%) had received thoracic radiotherapy (RT) for the treatment of NSCLC prior to the first cycle of pembrolizumab. Pembrolizumab was administered as monotherapy in 69 patients (67.6%) and in combination with chemotherapy in 33 patients. Pembrolizumab was used as first-, second-, and third-line or later therapy in 49 (48.0%), 32 (31.4%), and 21 (20.6%) patients, respectively. At the time of data cutoff, 20 patients (19.6%) were still receiving pembrolizumab treatment. Molecular analyses revealed epidermal growth factor receptor and ALK mutations in 6 of 101 and 1 of 87 cases, respectively.

**Table 1:** Baseline clinical characteristics of the study population at the initiation of pembrolizumab therapy.

Characteristics	No. (N = 102)
**Age median (range), yr**	69 (46–86)
**Sex**	
Male, n (%)	88 (86.3)
Female, n (%)	14 (13.7)
**Smoking**	
Never, n (%)	13 (12.7)
Former, n (%)	47 (46.1)
Current, n (%)	42 (41.2)
**ECOG performance status (PS)**	
1, n (%)	67 (65.7)
2, n (%)	30 (29.4)
3, n (%)	5 (4.9)
**Histology**	
Squamous cell carcinoma, n (%)	36 (35.3)
Adenocarcinoma, n (%)	59 (57.8)
Others non-small cell carcinoma, n (%)-Sarcomatoid carcinoma (n = 2), Unclassified non-small cell carcinoma (n = 5)	7 (6.9)
**PD-L1 (22C3)**	
<1%, n (%)	10 (9.8)
1%≥, <50%, n (%)	23 (22.6)
50%≥, n (%)	69 (67.6)
**Absolute neutrophil count** (/μL)	
<3165.9, n (%)	21 (20.6)
≥3165.9, n (%)	81 (79.4)
**Neutrophil–lymphocyte ratio**	
<2.18, n (%)	21 (20.6)
≥2.18, n (%)	81 (79.4)
**Derived neutrophil-lymphocyte ratio**	
<1.60, n (%)	31 (30.4)
≥1.60, n (%)	71 (69.6)
**Monocyte–lymphocyte ratio**	
<0.29, n (%)	33 (32.4)
≥0.29, n (%)	69 (67.6)
**Platelet–lymphocyte ratio**	
<169, n (%)	39 (38.2)
≥169, n (%)	63 (61.8)
**Systemic immune inflammation index**	
<563.1, n (%)	28 (27.5)
≥563.1, n (%)	74 (72.5)
**Hemoglobin (g/mL)^†^**	
<12.0, n (%)	52 (51.0)
≥12.0, n (%)	50 (49.0)
**Albumin (g/dL)^†^**	
<3.5, n (%)	21 (20.6)
≥3.5, n (%)	81 (79.4)
**Lactate dehydrogenase (IU/L)^†^ (n = 70)**	
≤250, n (%)	31 (44.3)
>250, n (%)	39 (55.7)
**C-reactive protein (mg/dL)^†^ (n = 78)**	
≤0.5, n (%)	28 (35.9)
>0.5, n (%)	50 (64.1)
**M stage**	
1a, n (%)	35 (34.3)
1b, n (%)	27 (26.5)
1c, n (%)	40 (39.2)
**Prior thoracic radiotherapy**	
None, n (%)	76 (74.5)
Radiation, n (%)	26 (25.5)
**Lines of pembrolizumab therapy**	
First, n (%)	49 (48.0)
Second, n (%)	32 (31.4)
Third more, n (%)	21 (20.6)
**Treatment regimen**	
Pembrolizumab monotherapy, n (%)	69 (67.6)
Pembrolizumab with chemotherapy, n (%)	33 (32.4)

^†^Dichotomized by cutoff of normal value. ECOG, eastern cooperative oncology group. PD-L1, programmed death ligand 1. N, total number. n, sample number.

### 3.2 PD-L1 (22C3) IHC Expression

Of the 102 samples analyzed, PD-L1 TPSs were <1% in 10 patients (9.8%), 1–49% in 23 patients (22.6%), and ≥50% in 69 patients (67.6%). In our study, patients were stratified into two categories based on a PD-L1 TPS cutoff value of 50%. The association between PD-L1 expression and baseline clinicopathological characteristics is presented in Supplementary Table S1. Significant differences between the high (≥50%) and low (<50%) PD-L1 expression groups were observed for prior thoracic radiotherapy (*p* = 0.002), line of pembrolizumab therapy (*p* = 0.047), and treatment regimen (monotherapy vs. combination therapy) (*p* < 0.001). No significant differences in OS (*p* = 0.469) or PFS (*p* = 0.703) were observed based on PD-L1 expression levels (Supplementary Fig. S1).

### 3.3 CBC-IBs

**Figure 2:**
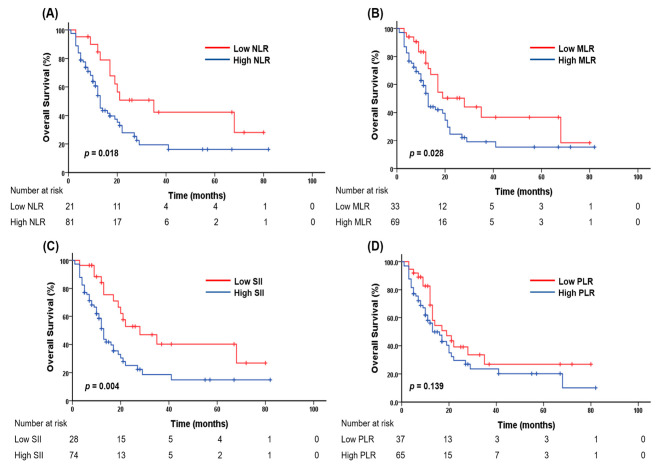
Kaplan–Meier curves for overall survival stratified by (**A**) neutrophil-to-lymphocyte ratio (NLR), (**B**) monocyte-to-lymphocyte ratio (MLR), (**C**) systemic immune-inflammation index (SII), and (**D**) platelet-to-lymphocyte ratio (PLR).

The optimal cutoff values for the ANC, NLR, dNLR, MLR, PLR, and SII were 3165.9 (1123.1–17,961.8), 2.18 (1.23–25.06), 1.60 (0.79–12.16), 0.29 (0.13–1.42), 168.8 (57.6–1144.3), and 563.1 (209.8–6739.9), respectively. Among these, NLR, dNLR, MLR, and SII values were identified as significant prognostic factors in the survival analysis. Lower NLR, dNLR, MLR, and SII values were significantly associated with improved OS. In particular, the median survival times (MSTs) for patients with low versus high values were 35 vs. 13 months for the NLR (*p* = 0.018), 21 vs. 13 months for the dNLR (*p* = 0.022), 28 vs. 13 months for the MLR (*p* = 0.028), and 28 vs. 13 months for the SII (*p* = 0.004; Fig. 2). However, in the multivariate analysis, only the SII remained independently associated with OS (*p* = 0.009; Table 2). High pretreatment PLR and SII values were significantly associated with poorer outcomes in terms of PFS. Patients with low versus high PLRs had a median PFS of 13 vs. 6 months (*p* = 0.036), whereas those with low versus high SII values had a median PFS of 13 vs. 6 months (*p* = 0.031). Notably, SII was associated with an even more substantial increase in the risk of disease progression than PLR, as reflected by a higher hazard ratio of 1.73 (Table 2, Supplementary Fig. S2). These findings indicate that lower pretreatment SII values were significantly associated with longer PFS and OS in patients treated with pembrolizumab compared to patients with high SII values. Among the evaluated CBC-IBs, SII exhibited the highest C-index for OS (0.562), followed by MLR (0.538), NLR (0.536), PLR (0.500), dNLR (0.500), ANC (0.476). Furthermore, SII was the only biomarker that maintained independent prognostic significance in the multivariate analysis, supporting its role as the most robust marker in this study.

**Table 2:** Univariate and multivariate analyses of predictive factors for overall survival and progression-free survival in patients receiving pembrolizumab treatment.

Characteristics	Overall Survival				Progression-Free Survival			
	Univariate Analysis		Multivariate Analysis		Univariate Analysis		Multivariate Analysis	
	HR (95% CI)	p*	AHR (95% CI)	p*	HR (95% CI)	p*	AHR (95% CI)	p*
**Age**		0.075				0.824		
≥70 (vs. <70)	1.56 (0.94–2.57)				1.05 (0.67–1.66)			
**Sex**		0.610				0.851		
Female (vs. male)	1.19 (0.60–2.37)				1.06 (0.57–1.97)			
**Smoking**		0.173				0.124		
For+cur (vs. never)	1.77 (0.76–4.14)				1.73 (0.88–3.63)			
**Histology**		0.424				0.402		
SQ (vs. Non-SQ)	1.23 (0.73–2.09)				1.21 (0.76–1.95)			
**ECOG PS**		0.285				0.442		
2–3 (vs. 0–1)	1.31 (0.79–2.18)				1.19 (0.75–1.91)			
**M stage**		<0.001		<0.001		<0.001		<0.001
M1c (vs. M1a–b)	1.93 (1.49–2.51)		3.25 (1.92–5.50)		1.67 (1.30–2.14)		2.79 (1.70–4.57)	
**PD-L1**		0.469				0.703		
<50% (vs. ≥50%)	1.24 (0.68–2.22)				1.10 (0.66–1.82)			
**Hemoglobin (g/mL)^†^**		0.050				0.668		
≤12 (vs. >12)	1.63 (0.98–2.70)				1.10 (0.70–1.73)			
**Albumin (g/dL)^†^**		0.054				0.269		
<3.5 (vs. ≥3.5)	1.72 (0.97–3.05)				1.33 (0.78–2.27)			
**CRP (mg/dL)^†^**		0.219				0.665		
>0.5 (vs. ≤0.5)	1.44 (0.79–2.64)				1.12 (0.65–1.94)			
**LDH (IU/L)^†^**		0.846				0.492		
>211 (vs. ≤211)	1.06 (0.58–1.95)				1.20 (0.69–2.08)			
**ANC** (/μL)		0.071				0.378		
≥3162.9 (vs. <3165.9)	1.81 (0.95–3.45)				1.29 (0.73–2.28)			
**NLR**		0.018		0.512		0.105		
≥2.18 (vs. <2.18)	2.15 (1.11–4.17)		1.41 (0.50–3.97)		1.59 (0.88–2.87)			
**dNLR**		0.022		0.965		0.052		
≥1.60 (vs. <1.60)	1.94 (1.10–3.42)		1.02 (0.41–2.55)		1.66 (1.00–2.77)			
**MLR**		0.028		0.241		0.102		
≥0.29 (vs. <0.29)	1.85 (1.05–3.28)		1.49 (0.77–2.90)		1.50 (0.90–2.48)			
**PLR**		0.139				0.036		0.654
≥168.8 (vs. <168.8)	1.48 (0.87–2.52)				1.65 (1.01–2.70)		1.16 (0.60–2.23)	
**SII**		0.004		0.009		0.031		0.434
≥563.1 (vs. <563.1)	2.34 (1.28–4.30)		2.37 (1.25–4.51)		1.73 (1.02–2.94)		1.32 (0.66–2.66)	
**Prior thoracic RT**		0.027		0.035		0.199		
None (vs. RT)	1.92 (1.06–3.49)		1.96 (1.05–3.66)		1.40 (0.82–2.38)			
**Lines of therapy**		0.346				0.578		
1st+2nd (vs. 3rd more)	1.31 (0.74–2.34)				1.16 (0.68–1.98)			
**Treatment regimen**		0.306				0.800		
Combine (vs. mono)	1.35 (0.75–2.46)				1.07 (0.64–1.77)			

†Dichotomized by cutoff of normal value. *p values denote statistical significance at the *p* < 0.05 level. Abbreviations: AHR, adjusted hazard ratio; ANC, absolute neutrophil count; CI, confidence interval; CRP, c-reactive protein; dNLR, derived neutrophil–lymphocyte ratio; ECOG PS, eastern cooperative oncology group performance status; For-cur, former-current; LDH, lactate dehydrogenase; MLR, monocyte–lymphocyte ratio; NLR, neutrophil–lymphocyte ratio; PD-L1, programmed death ligand 1; PLR, platelet–lymphocyte ratio; RT, radiotherapy; SII, systemic immune inflammation index.

### 3.4 Clinical Impact of the SII and PD-L1 Expression

At the time of the final analysis cutoff, 62 patients (60.8%) had died, and the MST of the entire cohort was 17 (95% CI: 12.8–21.2) months. These findings of the univariate and multivariate analyses of baseline clinical variables are summarized in Table 2. In the univariate analysis, the following variables were significantly associated with OS: M stage (*p* < 0.001), prior thoracic radiotherapy (*p* = 0.027), NLR (*p* = 0.018), dNLR (*p* = 0.022), MLR (*p* = 0.028), and SII (*p* = 0.004). Upon multivariate analysis, low SII (*p* = 0.009), M1a–b stage (*p* < 0.001), and prior thoracic radiotherapy (*p* = 0.035) remained independently associated with prolonged OS. Based on 75 events (73.5%) of disease progression or death, the median PFS was 7.0 (95% CI 4.9–9.1) months. In the univariate analyses, low SII (*p* = 0.031), low PLR (*p* = 0.036), and M1a–b stage (*p* < 0.001) were significantly associated with a longer PFS. However, in the multivariate analysis, only the M stage remained an independent predictor of disease progression (Table 2). The disease control rate (DCR) was 26.5% (27/102), showing the same value as the PFS rate. This is because all 75 patients categorized as PFS events, including 57 with progressive disease and 18 who died or were lost to follow-up prior to assessment, were consistently defined as non-responders for the DCR calculation.

**Figure 3:**
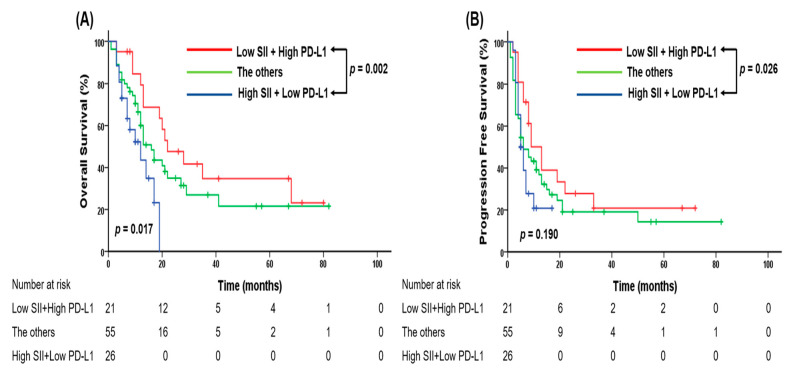
Kaplan–Meier curves for (**A**) overall survival and (**B**) progression-free survival according to three groups defined by the combined SII–programmed death ligand 1 (PD-L1) biomarker in patients with advanced non-small-cell lung carcinoma (NSCLC) treated with pembrolizumab.

To evaluate whether the SII could complement the limitations of PD-L1 expression as a predictive biomarker of the response to pembrolizumab, patients were stratified into three groups based on combined SII and PD-L1 status. Group A included patients with low SII values and high PD-L1 expression (n = 21), group B included those with high SII values and low PD-L1 expression (n = 26), and group C comprised all other cases (n = 55). Patients in group A demonstrated significantly longer OS and PFS compared to those in group B. In particular, the MST was 22 months in group A versus 12 months in group B (*p* = 0.002), whereas the median PFS was 13 months versus 5 months, respectively (*p* = 0.026; Fig. 3). Additionally, to further elucidate the clinical role of SII, we performed stratified analyses based on PD-L1 expression. Our findings revealed that the impact of SII on survival was particularly pronounced in the PD-L1 low subgroup, showing a significant association with OS (low vs. high SII, mean survival 32 M vs. MST 12 M, respectively, *p* = 0.007) but a less definitive correlation with PFS (low vs. high SII, MST 19 M vs. 5 M, respectively, *p* = 0.108).

## 4 Discussion

Our findings revealed that advanced NSCLC patients with low SII values before pembrolizumab therapy had significantly longer PFS and OS compared to those with high SII values. Of the CBC-IBs evaluated, SII demonstrated superior predictive value for immunotherapy outcomes. Furthermore, the combined biomarker analysis revealed that patients with low SII values and high PD-L1 expression exhibited more favorable OS and PFS outcomes than those with high SII values and low PD-L1 expression. These findings imply that the SII may complement the limitations of PD-L1 as a standalone predictive biomarker for immunotherapy response. In particular, SII and PD-L1 are widely used, readily available, and cost-effective biomarkers in clinical practice. To our knowledge, a comprehensive evaluation of CBC-IBs as predictive biomarkers in advanced NSCLC patients treated with pembrolizumab has not been previously reported.

Immunotherapy has emerged as a third-generation antitumor treatment, following cytotoxic chemotherapy and targeted therapy. The advent of targeted therapies and ICIs has revolutionized the treatment landscape of lung cancer. As a result of these therapeutic advances and a decline in incidence, mortality from NSCLC has decreased, and relative survival rates have improved over the past decade [21]. Despite the progress in targeted therapy, a significant proportion of lung cancer patients lack actionable oncogenic drivers. In these patients, immunotherapy has significantly improved survival outcomes and quality of life [22,23]. However, only a minority of patients experience durable clinical benefit from immunotherapy. For example, the 5-year survival rates reported in well-designed clinical trials for pembrolizumab monotherapy and pembrolizumab combined with chemotherapy in advanced NSCLC were 31.9% and 19.4%, respectively [23,24]. These findings emphasize the need to develop novel predictive biomarkers and to validate their use in guiding clinical decision-making for immunotherapy [25]. There remains an unmet need for reliable biomarkers that can identify patients likely to benefit from ICIs.

Knowledge of the association between cancer and inflammation dates back to the 19th century. Inflammation is now recognized as a hallmark of cancer that plays a pivotal role in all stages of tumor development and progression [8,9,10]. Recent studies have suggested that inflammation is a critical component of the tumor microenvironment and is associated with prognosis and therapeutic efficacy [26]. Hematologic inflammatory parameters such as neutrophils, lymphocytes, and platelets are used as markers of the systemic inflammatory response and play essential roles in the immune defense against tumor cells [27,28]. These CBC-IBs have been established as independent prognostic factors in various malignancies, including lung cancer [11,12,13,14,15]. Among CBC-IBs, the SII, which integrates peripheral neutrophil, lymphocyte, and platelet counts, may more comprehensively reflect the balance between host inflammatory and immune statuses; it has emerged as a promising predictor of tumor response and survival outcomes in patients undergoing cancer immunotherapy [11,12,13,29]. High SII, characterized by elevated platelets and neutrophils, reflects a potent immunosuppressive microenvironment. Specifically, platelets impair T-cell function via Transforming Growth Factor (TGF)-β signaling and physical shielding [30], while neutrophils suppress T-cell proliferation and Natural Killer (NK) cell cytotoxicity through Reactive Oxygen Species (ROS) production and PD-L1 upregulation [31]. Collectively, these components foster a ‘cold’ tumor environment that hinders T-cell infiltration and activation, thereby compromising the efficacy of PD-1 blockade and leading to poor clinical outcomes. 

Several studies comparing the prognostic performance of the SII with those of the NLR and PLR have demonstrated that the SII offers superior prognostic value in patients with lung cancer [14], these findings are consistent with our own, which showed that the SII outperformed the NLR, PLR, and MLR in predicting survival outcomes. This enhanced predictive capacity may be attributed to the inclusion of three immune-related parameters in the SII calculation. A recent meta-analysis indicated that elevated SII values are significantly associated with a poor prognosis and may be used as a reliable prognostic indicator in lung cancer patients receiving ICIs [11]. Consistent with these findings, we detected significant associations between elevated pretreatment SII values and poorer PFS and OS. However, in the multivariate analysis, SII remained an independent predictor only for OS. While the association with PFS did not reach statistical significance, a trend toward worse outcomes was observed (AHR = 1.32). This discrepancy may be because SII better reflects the host’s immune status and long-term survival potential rather than immediate radiographic response. And the lack of significance for PFS might stem from our limited sample size, patient heterogeneity, and the lack of a standardized SII cut-off. These results suggest that while SII is a robust indicator of long-term survival, its role in predicting immediate treatment response should be interpreted with caution. Moreover, while other established inflammatory indices (such as Lung Immune Prognostic Index (LIPI), Glasgow Prognostic Score (GPS) etc.) were excluded from this study due to missing baseline data, their clinical relevance cannot be overlooked. Future large-scale prospective studies are warranted to compare these markers with SII and further validate their integrated prognostic value.

Current National Comprehensive Cancer Network guidelines recommend upfront PD-L1 expression testing, which is classified as category 1 evidence, for patients with advanced NSCLC lacking actionable molecular biomarkers, regardless of histological subtype, before initiating first-line therapy. Pembrolizumab is listed as a preferred first-line treatment option in this setting. The choice between pembrolizumab monotherapy and combination therapy with chemotherapy depends on confirmed PD-L1 expression in tumor tissue [6]. To date, PD-L1 expression remains the most extensively studied and clinically validated biomarker for guiding immunotherapy in advanced NSCLC [7,32,33]. Notably, the FDA-approved PD-L1 IHC 22C3 pharmDx assay is a companion diagnostic for pembrolizumab [4]. However, several studies have raised concerns about the predictive value of PD-L1 expression alone. One study in PD-L1-positive advanced NSCLC found no significant difference in OS based on PD-L1 expression levels [34]. Another study involving patients with PD-L1 expression ≥50% reported estimated 6- and 12-month survival rates of 80% and 25%, respectively, under pembrolizumab therapy [3]. Moreover, some PD-L1-negative patients have been shown to respond to PD-1/PD-L1 inhibitors [35]. These findings imply that PD-L1 expression alone may be insufficient to predict immunotherapy responses reliably [36]. Consistent with these findings, our study demonstrated that PD-L1 expression alone was not a reliable indicator for selecting patients more likely to benefit from pembrolizumab therapy.

The limitations of PD-L1 expression as a standalone biomarker may be addressed by combining it with inflammatory parameters. A previous study demonstrated that combination of the pretreatment NLR and tumor mutational burden provided additional predictive value for the ICI response across several cancer types [37]. In our previous study, we observed that combination of the SII and PD-L1 expression was superior to PD-L1 alone in predicting survival and disease progression in patients with small-cell lung cancer treated with ICIs [12]. These findings support the hypothesis that CBC-IBs can compensate for the limitations of PD-L1 alone in identifying patients more likely to benefit from immunotherapy. Although PD-L1 expression alone did not demonstrate a statistically significant association with treatment outcomes in our study, the combined SII–PD-L1 biomarker exhibited significantly greater predictive value. In particular, patients in the low SII–high PD-L1 group exhibited significantly better survival and progression outcomes than those in the high SII–low PD-L1 group. In OS analysis according to SII after stratification by PD-L1 expression, the ability of SII to identify high-risk individuals within the PD-L1 low population highlights its value as a complementary biomarker. By integrating SII with PD-L1 status, clinicians may achieve more refined risk stratification, especially for patients who might otherwise be considered poor candidates for immunotherapy based on PD-L1 expression alone. Considering the practicality, low cost, and wide availability of SII and PD-L1 testing in clinical settings, we hypothesize that the combined SII–PD-L1 biomarker may be a more robust, clinically useful tool for predicting immunotherapy responses. 

As a single-center real-world preliminary study, our study has several limitations. First, the retrospective design may have introduced selection and information biases, although we attempted to minimize these by including all eligible patients with complete clinical and pathological information in our institutional database. Especially, our study may be subject to selection bias because we excluded patients who received fewer than 3 cycles of pembrolizumab. This criterion was necessary to ensure the availability of radiological response data and to minimize the confounding influence of pseudoprogression, as the first radiological evaluation at our institution is typically conducted after the third cycle. However, it may have led to the exclusion of rapid progressors or patients with early mortality, which could potentially result in an overestimation of survival outcomes. Second, this was a single-center study with a relatively small sample size, and the findings including the optimal cutoffs for inflammatory biomarkers have not been independently validated, limiting the generalizability of our findings. To mitigate these limitations and enhance the robustness of our analysis, we prospectively performed validated PD-L1 IHC analysis on diagnostic tumor samples for all included patients. Furthermore, all patients were accurately staged using standardized imaging modalities, including fluorodeoxyglucose PET-CT, brain imaging, and chest CT. To reduce potential confounding influences, we excluded patients with active infections, systemic inflammatory or autoimmune diseases, and those receiving medications known to affect CBC-IBs. We believe that the application of these rigorous inclusion and exclusion criteria, while potentially limiting the sample size, enhanced the quality and reliability of our findings. Our cohort was relatively heterogeneous, including patients receiving different treatment lines (first, second, and third lines and beyond) and regimens (monotherapy and chemoimmunotherapy). Although subgroup analyses for OS showed a significant association for SII in the monotherapy group (*p* = 0.028) and a consistent trend across other subgroups (*p*-values 0.070–0.094), the reduced sample size in each group may have limited our ability to achieve statistical significance. Future large-scale, prospective studies are warranted to validate the predictive power of SII across more homogeneous treatment settings. However, to ensure consistency, all baseline prognostic variables, including CBC-IBs, were reassessed immediately prior to the initiation of pembrolizumab therapy. Furthermore, to evaluate treatment responses accurately, we included only those patients who had received at least three cycles of pembrolizumab. 

## 5 Conclusion

In conclusion, our preliminary study in patients with advanced NSCLC demonstrated that higher pretreatment SII values were associated with poorer survival outcomes and a lower likelihood of response to pembrolizumab. These CBC-IBs are inexpensive, readily available, and easily measured in routine clinical practice before and during immunotherapy. Notably, the combination of pretreatment SII and PD-L1 expression provided additional prognostic and predictive value, offering more comprehensive guidance for treatment strategies in patients receiving pembrolizumab. Although further prospective and multicenter studies are needed to validate the generalizability of our findings, the clinical implication is that the use of pretreatment SII and/or PD-L1 expression values may predict therapeutic outcomes and assist in optimizing individualized treatment strategies for patients with advanced NSCLC. This preliminary study will be further extended according to the availability of datasets.

## Data Availability

The dataset supporting the conclusions of the current study is included within the article. The raw data generated and analyzed during the current study are not publicly available since they contain potentially identifying information. However, some raw datasets of the current study are available from the corresponding author on reasonable request.

## Supplementary Materials

The supplementary material is available online at https://www.techscience.com/doi/10.32604/or.2026.077514/s1.

## Abbreviations

ANC	Absolute neutrophil count
CBC-IB	Complete blood count-derived inflammatory biomarker
CI	Confidence interval
CRP	C-reactive protein
CT	Computed tomography
dNLR	Derived neutrophil–lymphocyte ratio
ECOG PS	Eastern Cooperative Oncology Group performance status
FDA	U.S. Food and Drug Administration
HR	Hazard ratio
ICI	Immune checkpoint inhibitor
IHC	Immunohistochemistry
LDH	Lactate dehydrogenase
MLR	Monocyte–lymphocyte ratio
MST	Median survival time
NLR	Neutrophil–lymphocyte ratio
NSCLC	Non-small-cell lung cancer
OS	Overall survival
PD-1/PD-L1	Programmed death-1 or its ligand 1
PET	Positron emission tomography
PFS	Progression-free survival
PLR	Platelet–lymphocyte ratio
RT	Radiotherapy
SII	Systemic immune-inflammation index
TPS	Tumor proportion score
