# Supplementary material for: Clinical Value of the Systemic Immune Inflammation Index and PD-L1 Expression in Advanced NSCLC Treated with Pembrolizumab: Real-World Preliminary Study
Source: Oncol Res. 2026 May 21;34(6):23. doi: 10.32604/or.2026.077514 (PMC13223186; doi:10.32604/or.2026.077514)
Supplement: Supplementary file 1 [file OncolRes-34-77514-s001.zip › TSP_OR_77514-s001.docx]

**Table S1**. Baseline clinical characteristics of the study population according to programmed death ligand 1 (PD-L1) expression, assessed using the 22C3 pharmDx assay.

| Variables | No.  *N* = 102 | PD-L1 (22C3) Immunohistochemistry Expression | | |
| --- | --- | --- | --- | --- |
|  |  | **< 50%** | **≥ 50%** | ***p* value*** |
| Age |  |  |  | 0.283 |
| <70, n (%) | 54 (52.9) | 20 (60.6) | 34 (49.3) |  |
| ≥70, n (%) | 48 (47.1) | 13 (39.4) | 35 (50.7) |  |
| Sex |  |  |  | 0.999 |
| Male, n (%) | 88 (86.3) | 29 (87.9) | 59 (85.5) |  |
| Female, n (%) | 14 (13.7) | 4 (12.1) | 10 (14.5) |  |
| EGOG PS |  |  |  | 0.300 |
| 0-1, n (%) | 67 (65.7) | 24 (72.7) | 43 (62.3) |  |
| 2-4, n (%) | 35 (34.3) | 9 (27.3) | 26 (37.7) |  |
| Smoking |  |  |  | 0.752 |
| Current+Former, n (%) | 89 (87.3) | 28 (84.8) | 61 (88.4) |  |
| Never, n (%) | 13 (12.7) | 5 (15.2) | 8 (11.6) |  |
| Histology |  |  |  | 0.876 |
| SQ, n (%) | 36 (35.3) | 12 (36.4) | 24 (34.8) |  |
| Non-SQ, n (%) | 66 (64.7) | 21 (63.6) | 45 (65.2) |  |
| ANC (/μL) |  |  |  | 0.144 |
| < 3165.9, n (%) | 21 (20.6) | 4 (12.1) | 17 (14.2) |  |
| ≥ 3165.9, n (%) | 81 (79.4) | 29 (87.9) | 52 (54.8) |  |
| NLR |  |  |  | 0.678 |
| < 2.18, n (%) | 21 (20.6) | 6 (18.2) | 15 (21.7) |  |
| ≥ 2.18, n (%) | 81 (79.4) | 27 (81.8) | 54 (78.3) |  |
| dNLR |  |  |  | 0.350 |
| < 1.60, n (%) | 31 (30.4) | 8 (24.2) | 23 (33.3) |  |
| ≥ 1.60, n (%) | 71 (69.6) | 25 (75.8) | 46 (66.7) |  |
| MLR |  |  |  | 0.293 |
| < 0.29 , n (%) | 33 (32.4) | 13 (39.4) | 20 (29.0) |  |
| ≥ 0.29, n (%) | 69 (67.6) | 20 (60.6) | 49 (71.0) |  |
| PLR |  |  |  | 0.547 |
| < 168.8 , n (%) | 39 (38.2) | 14 (42.4) | 25 (36.2) |  |
| ≥ 168.8, n (%) | 63 (61.8) | 19 (57.6) | 44 (63.8) |  |
| SII |  |  |  | 0.329 |
| < 563.1 , n (%) | 28 (27.5) | 7 (21.2) | 21 (30.4) |  |
| ≥ 563.1, n (%) | 74 (72.5) | 26 (78.8) | 48 (69.6) |  |
| Hemoglobin(g/mL)^†^ |  |  |  | 0.232 |
| < 12.0, n (%) | 52 (51.0) | 14 (42.4) | 38 (55.1) |  |
| ≥ 12.0, n (%) | 50 (49.0) | 19 (57.6) | 31 (44.9) |  |
| Albumin (g/dL) ^†^ |  |  |  | 0.914 |
| < 3.5, n (%) | 21 (20.6) | 7 (21.2) | 14 (20.3) |  |
| ≥ 3.5, n (%) | 81 (79.4) | 26 (78.8) | 55 (79.7) |  |
| LDH (IU/L) ^†^ |  |  |  | 0.313 |
| ≤ 250, n (%) | 31 (44.3) | 14 (51.9) | 17 (39.5) |  |
| > 250, n (%) | 39 (55.7) | 13 (48.1) | 26 (60.5) |  |
| CRP (mg/dL) ^†^ |  |  |  | 0.806 |
| ≤ 0.5, n (%) | 28 (35.9) | 12 (37.5) | 16 (34.8) |  |
| > 0.5, n (%) | 50 (64.1) | 20 (62.5) | 30 (65.2) |  |
| M stage |  |  |  | 0.372 |
| 1 a-b, n (%) | 62 (60.8) | 18 (54.5) | 44 (63.8) |  |
| 1 c, n (%) | 40 (39.2) | 15 (45.5) | 25 (36.2) |  |
| Prior thoracic RT |  |  |  | 0.002 |
| No-RT, n (%) | 76 (74.5) | 31 (93.9) | 45 (65.2) |  |
| RT, n (%) | 26 (25.5) | 2 (6.1) | 24 (34.8) |  |
| Lines of therapy |  |  |  | 0.047 |
| 1^st^ +2^nd^, n (%) | 81 (79.4) | 30 (90.9) | 51 (73.9) |  |
| 3^rd^ more, n (%) | 21 (20.6) | 3 (9.1) | 18 (26.1) |  |
| Treatment regimen |  |  |  | < 0.001 |
| Mono, n (%) | 69 (67.6) | 4 (12.1) | 65 (94.2) |  |
| Combine, n (%) | 33 (32.4) | 29 (87.9) | 4 (5.8) |  |

Note: ^†^Dichotomized by cutoff of normal value. * p values denote statistical significance at the p < 0.05 level. ANC, absolute neutrophil count; CRP, c-reactive protein; dNLR, dereived neutrophil–lymphocyte ratio; ECOG PS, Eastern Cooperative Oncology Group performance status; LDH lactate dehydrogenase; MLR, monocyte–lymphocyte ratio; NLR, neutrophil–lymphocyte ratio; PLR, platelet–lymphocyte ratio; RT, radiotherapy; SII, systemic immune inflammation index. N, total number; n, sample number.


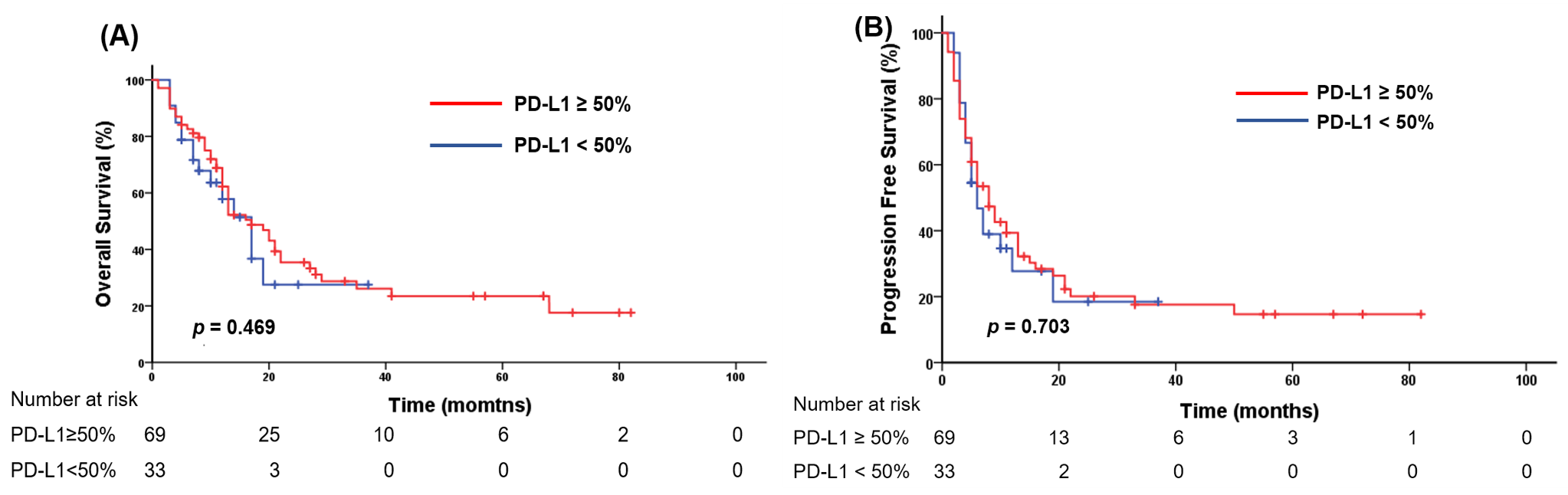
**Figure S1**. Kaplan–Meier curves for (A) overall survival and (B) progression-free survival according to *PD-L1 (22C3)* expression status.


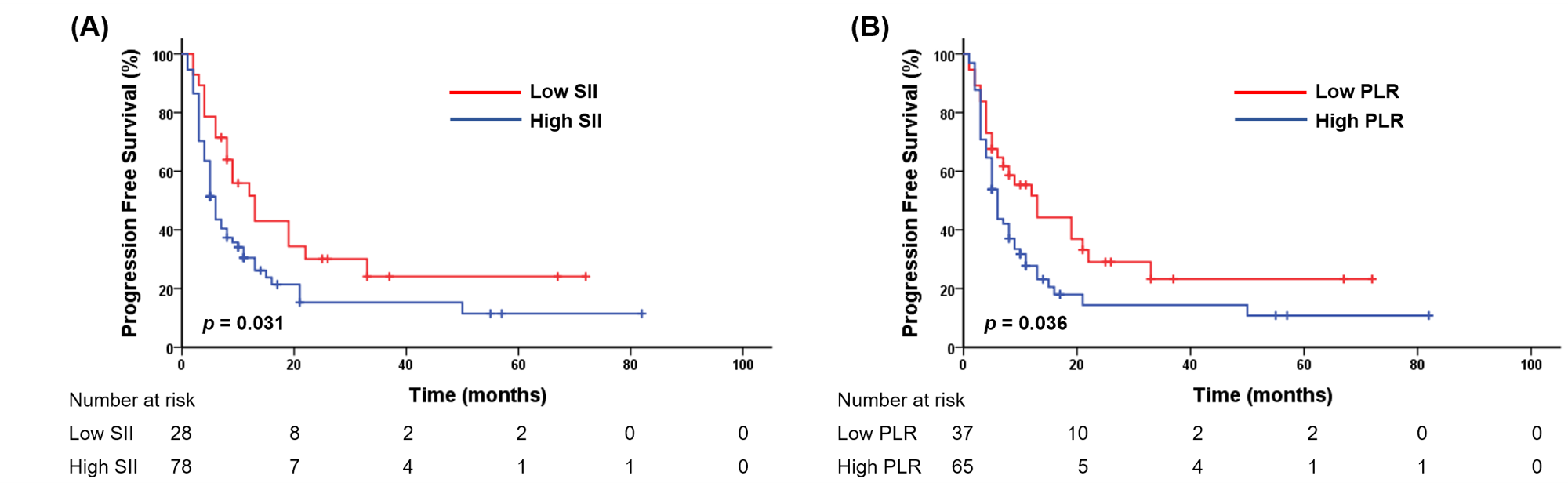


**Figure S2.** Kaplan–Meier curves for progression free survival stratified by (A) systemic immune-inflammation index (SII) and (B) platelet-to-lymphocyte ratio (PLR).
